# Determinants of Noninvasive Ventilation Outcomes during an Episode of Acute Hypercapnic Respiratory Failure in Chronic Obstructive Pulmonary Disease: The Effects of Comorbidities and Causes of Respiratory Failure

**DOI:** 10.1155/2014/976783

**Published:** 2014-01-19

**Authors:** Angela Maria Grazia Pacilli, Ilaria Valentini, Paolo Carbonara, Antonio Marchetti, Stefano Nava

**Affiliations:** ^1^Alma Mater Studiorum, Department of Specialistic, Diagnostic and Experimental Medicine (DIMES), University of Bologna, 40138 Bologna, Italy; ^2^Respiratory and Critical Care, Sant'Orsola Malpighi Hospital, Alma Mater Studiorum, Department of Specialistic, Diagnostic and Experimental Medicine (DIMES), University of Bologna, 40138 Bologna, Italy

## Abstract

*Objectives*. To investigate the effect of the cause of acute respiratory failure and the role of comorbidities both acute and chronic on the outcome of COPD patients admitted to Respiratory Intensive Care Unit (RICU) with acute respiratory failure and treated with NIV. *Design*. Observational prospective study. *Patients and Methods*. 176 COPD patients consecutively admitted to our RICU over a period of 3 years and treated with NIV were evaluated. In all patients demographic, clinical, and functional parameters were recorded including the cause of acute respiratory failure, SAPS II score, Charlson comorbidity index, and further comorbidities not listed in the Charlson index. NIV success was defined as clinical improvement leading to discharge to regular ward, while exitus or need for endotracheal intubation was considered failure. *Results*. NIV outcome was successful in 134 patients while 42 underwent failure. Univariate analysis showed significantly higher SAP II score, Charlson index, prevalence of pneumonia, and lower serum albumin level in the failure group. Multivariate analysis confirmed a significant predictive value for pneumonia and albumin. *Conclusions*. The most important determinants of NIV outcome in COPD patients are the presence of pneumonia and the level of serum albumin as an indicator of the patient nutritional status.

## 1. Introduction

Patients with acute respiratory acidosis caused by an exacerbation of chronic obstructive pulmonary disease (COPD) are the group that benefits most from noninvasive ventilation [1]. Its early use in patients with COPD who have mild respiratory acidosis (as low as pH 7.30) and mild-to-moderate acute respiratory failure (ARF) prevents further deterioration and thus avoids endotracheal intubation and improves survival compared with standard medical therapy [2]. “Real life” observational studies, performed outside specific RCTs, have shown on average a higher percentage of NIV failure than those performed in selected population [3, 4]. Most of the RCTs have enrolled, as a matter of fact, very selected population of patients. In particular subjects with some comorbidities like pneumonia, neurological and cardiac diseases, dementia, myocardial infarction, and severe obesity were excluded a priori from those studies [2, 5–7]. A recent study pointed out that COPD is not very often a “stand-alone” disease since comorbidities are frequent and 12 of them negatively influence the 4-year survival [8]. Whether the presence of chronic comorbidities could also influence the short-term mortality and the NIV success during an acute exacerbation of COPD was only partially investigated. Scala et al. in 2004 [9] showed that the presence of acute and chronic comorbidities negatively influences NIV outcome. On the other hand some acute comorbidities,” such as pulmonary oedema, may per se cause of ARF [10]. At present little data is available regarding the “individual” effect of comorbidities, since their analysis was mainly performed as a group one (i.e., cardiovascular, noncardiovascular, metabolic, etc). In the present observational study we wanted to investigate the possible effect on NIV outcomes of (1) the etiology of ARF and (2) the overall number and “individual” chronic and acute comorbidities in COPD patients ventilated for an acute exacerbation of their disease.

## 2. Material and Methods

In an analysis of data collected prospectively and inserted in a database, we evaluated data collected from 176 consecutive patients with COPD exacerbation admitted over 36-month period to our 7-bed Respiratory Intensive Care Unit (RICU). COPD diagnosis was based on pulmonary function tests when available, otherwise on clinical history, physical examination, and imaging data (chest radiograph or HRCT scan) according to the 1987 ATS statement [11].

The protocol of the study was approved by the scientific committee of our institution. The ethical committee commented that the approval was waived since the patients at hospital admission must sign or not the consent that their medical records and “routine” examinations may be used for research proposals. We have analyzed only records of patients who agreed to sign.

Inclusion criteria were pH < 7.35 and a PaCO_2_ > 45 mmHg with at least one of these three criteria: massive use of accessory muscles, respiratory rate > 20 breaths/min, and severe dyspnoea (Borg ≥ 5).

Exclusion criteria were (a) refusal of NIV; (b) facial deformity sufficient to affect mask fitting; (c) overt gastrointestinal bleeding; (d) upper airway obstruction; (e) acute ischaemic heart disease; (f) need for urgent intubation due to cardiac or respiratory arrest, prolonged respiratory pauses, and psychomotor agitation requiring sedation; (g) ventricular arrhythmia requiring treatment; (h) cerebrovascular accident. Postoperative patients were also excluded. All the patients first underwent standard medical therapy lasting about 45–60 min before initiating a trial of NIV.

### 2.1. NIV Settings

Patients were ventilated with pressure support ventilation (PSV) or pressure-controlled ventilation (PCV) using a full face mask or the helmet in few cases. The inspiratory pressure was adjusted according to the patient's tolerance to obtain an expired tidal volume of 7-8 mL/kg with external PEEP not exceeding 6 cmH_2_O. ICU ventilators using NIV mode or dedicated NIV platforms (all of which allowed exhaled tidal volume to be recorded) were used. Flow and pressure curves were used to detect the possible occurrence of patient/ventilator mismatching. Trigger sensitivity was set usually at 5 L/min or the one imposed by the machine was used if setting was not possible. FiO_2_ was set to achieve an SaO_2_ of >90% and not >95%.

### 2.2. Definitions of NIV Success or Failure

Success was defined as the achievement of a clinical and functional condition stable enough to allow patient discharge to the ward.

Failure was defined, at any time during the RICU stay, as a sudden or progressive worsening of arterial blood gas tensions (pH < 20% of the last arterial blood gases with an increase in PaCO_2_ of >15–20% compared with previous arterial blood gas tensions), dyspnea, and/or sensory deterioration while still on mechanical ventilation for at least 6 hours/day. Death while in the RICU was also considered as NIV failure.

The need of intubation in the failure group was decided by the attending physician, always according to our internal guidelines and/or clinical judgments and upon the criteria of failure above reported.

### 2.3. Measurements

The following variables were recorded in all patients: age, sex, SAPS II score severity illness defined as the worst value measured within the first 24 hours of RICU admission, Barthel index, blood gases analysis values at the admission, 2 hours after NIV institution, and at the time of RICU discharge (success group) or prior to intubation or death (failure group), and length of hospital stay before RICU admission.

Causes of acute respiratory failure were classified as follows: (1) pneumonia, (2) pulmonary embolism, (3) pneumothorax, (4) congestive heart failure, and (5) cardiogenic pulmonary edema.

Types and number of acute [12] and chronic [13] nonrespiratory comorbidities according to the Charlson index were recorded at hospital admission.

Other causes of chronic disorders, not included in the Charlson index, were also recorded, such as obesity (BMI > 35), diagnosed overlap syndrome, dysfunction of the diaphragm (assessed through imaging methods such as standard chest X rays, CT, and sniffing test, revealing elevation and hypomobility), pneumonectomy, and bed-ridden syndrome.

The primary end points of the study were to detect differences, if any, in the clinical variables at admission, in the two groups of patients (NIV success or failure), and to eventually depict a priori predictors of the NIV outcome.

### 2.4. Statistical Analysis

Descriptive statistics are expressed as mean and standard deviation (SD) for continuous variables and percentage for categorical variables. Demographic and comorbidities characteristics were compared among the two groups (NIV success or failure) on continuous variables using *t*-tests, on ordinal-level variables using the Mann-Whitney test, and on categorical variables using *χ* ^2^ tests or Fisher exact test, as appropriate.

Univariate and multivariate analysis and odds ratio (OR) were estimated with logistic regression for identifying the risk factors associated with NIV outcome, using the clinical variables illustrated in the Material and Methods. In particular a set of variables significantly associated with success in univariate analyses were included in a multiple logistic regression analysis. A *P* value less than 0.05 was considered statistically significant. All statistical analyses were carried out using IBM SPSS Statistical Software, version 20.0.

## 3. Results

**Figure 1:**
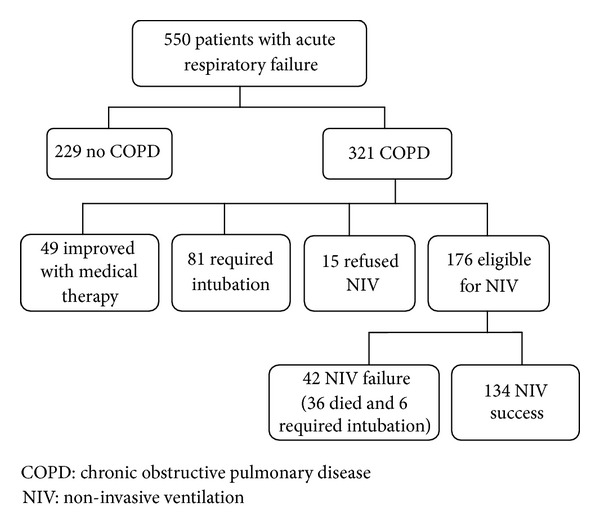
Patients flow through the study.

Figure 1 illustrates the patients flow through the study. A total of 550 patients were admitted in our RICU during the time period considered. Of these, 321 had COPD with acute or acute on chronic respiratory failure; the remaining 229 cases included patients without COPD and with respiratory failure caused by different conditions; 15 patients intolerant to NIV in the first hour of treatment were excluded from data analysis; 49 patients required oxygen therapy alone, while 81 required invasive ventilation (either via endotracheal tube or tracheostomy). A total of 176 patients were enrolled in the study; 134 were successfully ventilated with NIV and discharged alive, and 42 were considered NIV failure (36 deaths and 6 intubation). Causes of death in those patients (some patients presented more than one cause) were cardiogenic pulmonary edema (7), pneumonia (12), sepsis (8), atrial or ventricular arrhythmia (11), myocardial infarction (2), intestinal occlusion (1), pneumothorax (1), and lung cancer (2).

Overall patients characteristics' at the time of RICU admission are illustrated in Table 1. Patients failing NIV were significantly older, sicker according to the SAPS II score and albumin level, and with a lower degree of independence, as assessed by the Barthel index. Length of hospital stay before admission in RICU was also higher in the failure group.

Interestingly arterial blood gases (ABGs) on admission were not statistically different between the two groups.

Table 2 shows the causes of ARF. Those patients failing NIV had a significantly higher number of pneumonia than those who succeeded.

Table 3 reports the numbers and types of acute and chronic comorbidities in the two groups of patients. Chronic heart failure was prevalent in both groups.

Dementia was significantly higher in the failure group. The presence of chronic renal failure was also higher in the failure group, although the difference did not achieve a level of statistical significance (*P* = 0.06).

**Figure 2:**
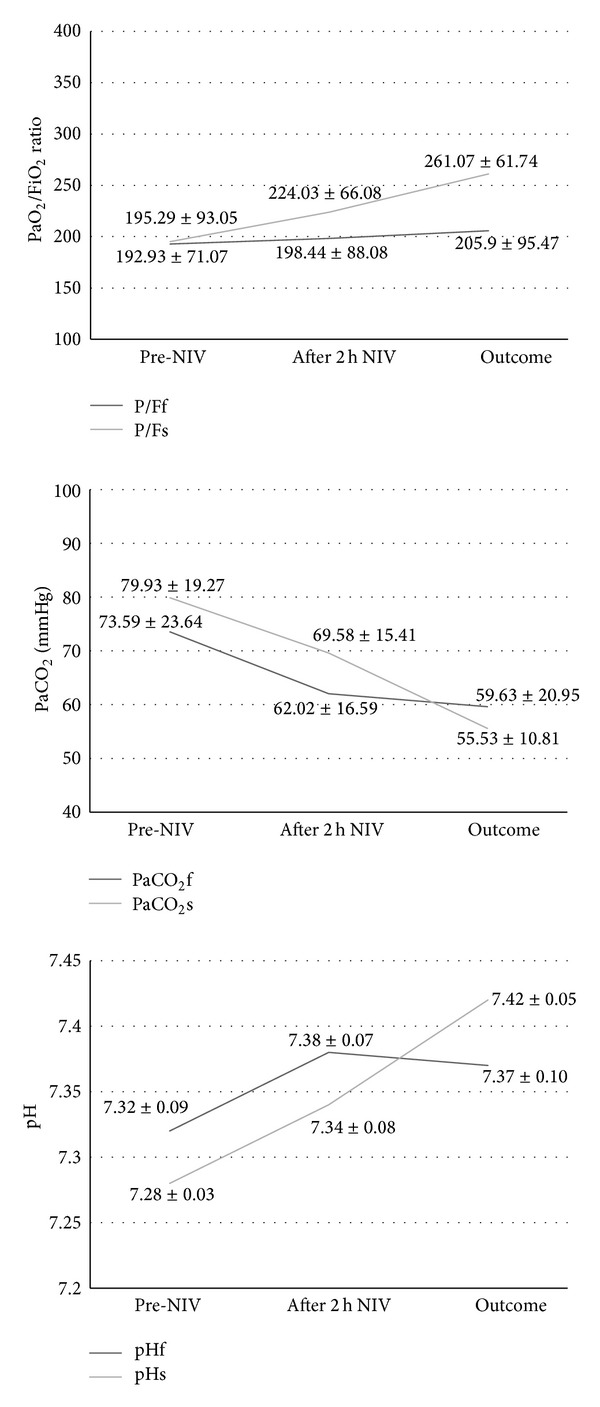
Arterial blood gases (ABGs) changes during the time course of the study. Outcomes mean discharge from the Respiratory Intensive care Unit (RICU) in the success group and death or time of intubation in the failure one.

Overall, the number of chronic comorbidities was significantly higher in the failure group versus the successful one (Charlson score 4.12 ± 2.15 versus 3.46 ± 1.6, resp., *P* = 0.035).  Figure 2 shows the ABGs changes after 2 hours of NIV and at the last measurement in the two groups. Interestingly, NIV failure group had a statistically higher pH on admission; they still had a significantly higher pH and also a lower PaCO_2_ level after 2 hours of ventilatory treatment compared to the patients who succeeded, despite that the percentage changes were similar in the two groups. The level of blood bicarbonates was equally high at the onset in both subgroups (34.9  mMol/L and 34.03  mMol/L in the failure group and in the successful group, resp.; difference is not significant) with no significant changes after 2 hours of NIV; however, at the time of discharge the success group had significantly higher bicarbonates than the failure group (36 mMol/L versus 33.4  mMol/L, *P* = 0.03). The PaO_2_/FiO_2_ ratio was not different between the two groups. At the time of discharge from the RICU the success group had statistically better gas exchange parameters versus those patients who required intubation or died.

Four variables were significantly associated with NIV success on univariate analysis (SAPS II score, Charlson score, serum albumin, or presence of pneumonia) and were eligible to be entered in the multivariate analysis in addition to age, gender, and renal failure, which were almost statistically significant.

Table 4 shows that serum albumin and pneumonia retained a significant predictive value; in particular, probability of success increases by 5.6 times for every 1 g/dL increase in albumin serum level, while presence of pneumonia decreases the success probability by 61.8%.

## 4. Discussion

This observational “real life” study showed that the cause of ARF, pneumonia in particular, is the main determinant of NIV success in COPD patients, rather than the presence of comorbidities.

It is well known that many patients dying do so during a severe COPD exacerbation, when they experience acute respiratory failure [14]. There is general agreement that NIV may prevent further deterioration in gas exchange, dyspnoea, respiratory workload, and endotracheal intubation in patients hospitalized for exacerbations of COPD with rapid clinical deterioration [1, 2]. Unfortunately a considerable number of the patients receiving NIV (approximately 30%) still require endotracheal intubation or die [4].

Several studies have assessed possible determinants of NIV failure in COPD patients, and most of them concluded that the severity of gas exchange (i.e., PaCO_2_ and pH in particular) at admission and its changes after 1 or 2 hours of ventilation are the major predictors of patients' outcome [12, 15].

More comprehensive risk chart for NIV failure has been also proposed including also other clinical parameters such as respiratory rate, severity score, and neurological status [15]. All these parameters are good markers of severity of the episode of ARF, and they may drive for sure the decision of the clinician to go further with NIV or pass directly to invasive ventilation, but they are overall epiphenomena of one or more underlying diseases. Surprisingly only very few studies have included in their analysis the importance of the cause of ARF [3] and/or the potential effect of the chronic comorbidities [9] that very often are present in COPD patients.

Concerning the cause of acute exacerbation, Seneff et al. [10] have demonstrated that they are not only necessarily related to a “simple” respiratory infection, but also to pneumonia, cardiovascular problems, pulmonary embolism, and pneumothorax. Concerning the effect of comorbidities, Divo et al. [8] have recently reported that easily identifiable comorbidities confer an independent risk of death when the patients are still in a phase of clinical stability. A large cross-sectional study performed in the USA has shown that, among other variables, more comorbidities conditions were independent risk factors for in-hospital mortality [16].

Only one study [9] so far investigated to our knowledge the role of comorbidities in determining NIV failure during an exacerbation of COPD. It divided the types of comorbidities into chronic or acute, and their prevalence was significantly higher in the NIV failure group. No study to our knowledge has so far attempted to assess the individual power of single comorbidities. The present study shows clearly that the main independent determinant of NIV failure was the presence of pneumonia at admission to the hospital, as a cause of ARF, and it accounted for >45% of these patients. Persons with comorbidities, COPD in particular, and elderly persons are at increased risk of pneumonia and of having a more complex illness [17]. The patients failing NIV were in our study on average significantly older and malnourished since their albumin level was lower, and, in this subset of individuals, mortality reached up to 30% [18].

In agreement with our data, the presence of pneumonia has been associated already as a predictor of NIV failure, together with lack of improvement of pH [3]. On the contrary in a randomized controlled trial [19] performed on selected patients with ARF caused by severe community-acquired pneumonia (CAP), NIV was associated when compared to medical treatment with a significant reduction in the rate of endotracheal intubation (zero percent) and duration of ICU stay only in the subgroup of COPD patients. The difference between the studies relies probably not only on the study design (RCT versus observational), but also mostly on the different population of patients; those in Confalonieri's study [19] were much younger and likely less severe. As of interest the presence of pneumonia was considered, in most of the RCTs performed in COPD, an exclusion criterion for the enrolment of patients, and this explains why very little attention has been paid so far to the “negative” predicted role of pneumonia in determining NIV success. In the present paper, due to difficulties in picking up the exact timing of diagnosis (ER stay before admission, etc.) we were unable to discriminate between CAP and HCAP, so we considered in the analysis the overall presence of pneumonia.

Overall the high prevalence of chronic comorbidities found in our population is in line with most of the previous studies performed during an exacerbation of COPD (42–97%) [20–22] but higher than in Scala's study [9], probably once more due to the older age of our sample and also the fact that, in Charlson index, COPD is per se accounted for as one of the comorbidities, and therefore all the enrolled patients scored at least 1 point, while this was not the case of the above mentioned investigation.

In general, as shown in Table 5, comparisons between the different studies are difficult on account of the heterogeneity in the patients populations in terms of age, the setting of the study (RICU [12], respiratory monitoring unit in a respiratory ward [9], ICU [10, 20], both hospital ward and ICU [22]), type of ventilatory treatment (noninvasive [9, 12, 20], invasive [10], no mechanical ventilation [21]), criteria used to assess severity (APACHE II [12, 20], APACHE III [9, 10, 22]), and, not least, the criteria employed to evaluate comorbidities (Charlson score [9], comorbidities included in the APACHE II [20] and III [10] scores, classes of comorbidities, e.g., cardiovascular and noncardiovascular [9], cardiac, pulmonary, metabolic, renal, gastrointestinal [12], etc.).

Among the Charlson comorbidities, the most prevalent in our study (obviously besides the chronic respiratory disease) was congestive heart failure (overall 88%, subgroups 93% and 86%, resp.) followed by renal failure (overall 18,2%, subgroups 28,6% and 15%); the prevalence of the other comorbidities was much lower.

The number of comorbidities was higher in our NIV failure group versus those patients who succeeded, but the predictive value of the Charlson index was very weak in the multivariate analysis and was not accounted for in the multivariate one. This is perfectly in agreement with Seneff et al. who demonstrated that the number of preexisting comorbidities was not a significant predictor of in-hospital mortality among patients admitted to the ICU [10]. In both studies the number of comorbidities itself may not have provided additional predictive power since we have examined a stratum of patients already at an elevated risk of death and not a broader range of disease severity due to the nature of our study performed in a RICU.

Interestingly some variables were statistically different in the two groups of patients. Dementia has a high prevalence in old ICU patients, most often associated with lack of physician awareness [23], and in our study was more prevalent in the failure group. Pisani et al. [23] documented no difference in outcomes from ICU care in older patients with and without dementia. Physical consequences of dementia predispose patients to infection, especially aspiration pneumonia [24, 25] and urinary tract infections [26]. This may have predisposed these patients to develop pneumonia that was therefore the leading cause of ARF. Another possibility of the higher percentage of patients in the NIV failure group is the lack of tolerance to NIV, although those patients intolerant to ventilation were excluded a priori from the analysis. Indeed our nursing team is specialized and trained to offer continuous care and support even for unconscious patients undergoing NIV.

Two rather common comorbidities were not associated with poor outcome of our patients. First obesity was surprisingly more prevalent in the success group. Many epidemiological studies have demonstrated that obesity is associated with higher morbidity and mortality rates in the general population [27, 28], but a more recent study demonstrated that BMI did not have a significant impact on mortality in ICU patients [29]. Obesity has been reported to increase respiratory muscle oxygen demand, with more oxygen being consumed for any given task compared to patients with normal weight, especially when undergoing an episode of ARF, but they are also likely to respond faster to NIV than expected [30].

About 20% of the whole group of patients had complicated or noncomplicated diabetes, but this was not a determinant of success or failure. We have shown [12] previously that “late NIV failure” defined by deteriorating gas exchange was more frequent in patients with an initially raised blood sugar. More recently, in a relatively small study, Chakrabarti et al. [31] found that hyperglycaemia, even when defined at only one time point, was related to the final NIV outcome irrespective of the diagnosis of diabetes, use of insulin, or prior oral corticosteroid use. Unfortunately the timing of our recording of blood glucose was not standardized so that it was impossible to get “unbiased” data, since the level of glycaemia is affected by fasting and actual medical therapy. Indeed, also for the above mentioned reason, hyperglycaemia cannot be considered a comorbidity, but rather an indicator of distress.

Use of domiciliary NIV did not influence the outcome in our patients (among the failure group, 14.3% of patients were on home NIV versus 11.2% cases in the success group; difference is not significant).

Last, surprisingly in this investigation we were unable to demonstrate a predictive effect of ABGs on the NIV outcome. As a matter of fact the patients who successfully responded had a significantly lower pH on admission and retained a lower pH after 2 hours of NIV. At first sight the results are not in line with the findings of previous reports [15]. It is likely that in elderly patients with multiple comorbidities the pathogenesis of acidosis is multifactorial and this may account for the apparent worse initial response to ventilator treatment in the group that in the end fared better. The relatively low degree of acidosis (pH > 7.26 in both groups) may also explain the initial homogeneous improvement in both groups. The level of blood bicarbonates in the two groups was not significantly different at the onset and after 2 hours of NIV and was elevated, likely because in most cases the patients had already an underlying chronic respiratory failure, and also because in ER or even during ambulance transport to the hospital they may have been administered i.v. bicarbonates to buffer the acidosis; however the difference became significant at the last measurement, with the failure group exhibiting lower levels, perhaps indicating an earlier exhaustion of the kidney bicarbonate reabsorption capacity in these patients.

The difference in gas exchange became significant at the time of intubation or death (failure) versus discharge from the RICU (success), and it was particularly true for the PaO_2_/FiO_2_ ratio. This seems to indicate that the improvement in such complicated patients is not always apparent at the beginning of the ventilator treatment but occurs over a longer period of time during the RICU stay, making it more difficult to predict the outcome after only a few hours.

## 5. Conclusions

Our study has some important limitations. First, it is a single-center study, and therefore it may reflect a specific reality where the NIV team is skilled and used to managing a large number of patients/year, and therefore these data may not be generalized. Second, only a minority of patients obtained the microbiological origin of their pneumonia, so the different pathogens may have affected the outcome of the patients. Third, for some patients pulmonary function tests data was not available and the diagnosis of COPD was therefore clinical/radiological. Last, the criteria of intubation were not standardized a priori, as for an RCT, but we followed our institutional guidelines about this, and on the other hand we are confident that our data reflect the “real life” scenario, rather than the “selected world” of those mentioned studies.

In conclusion, in this observational study we have shown that, in an unselected group of patients with ARF due to COPD exacerbation, the number of chronic comorbidities is quite elevated, but we were unable to discriminate between those patients who succeed or fail an NIV trial. The cause of ARF, pneumonia in particular, is the major independent discriminant of NIV failure, while ABGs in this study were not initial indicators of success or failure.

## Figures and Tables

**Table 1:** Differences in the main demographic clinical and functional parameters between the two groups of patients. Values are expressed as mean ± standard deviation. SAPS: simplified acute physiology score, ABG: arterial blood gases, NIV: noninvasive ventilation, RICU: Respiratory Intensive Care Unit, LTOT: long term oxygen therapy, and HMV: home mechanical ventilation.

	42 NIV-failure	134 NIV-success	*P* value
Age (years)	81.05 ± 7.05	77.81 ± 9.27	**0.019**
Albumin (g/dL)	3.07 ± 0.48	3.54 ± 0.46	**0.047**
SAPS score	44.50 ± 10.24	37.28 ± 8.87	<**0.001**
Barthel score	7.95 ± 9.37	15.86 ± 17.77	<**0.001**
ABGs admission			
P/F (PaO_2_/FiO_2_ ratio)	193 ± 93.05	195 ± 71.07	0.862
PaCO_2_ (mmHg)	73.60 ± 23.64	79.93 ± 19.27	0.080
pH	7.32 ± 0.09	7.28 ± 0.09	**0.015**
Days in hospital before RICU admission	9.21 ± 13.93	4.59 ± 8.79	**0.047**
LTOT	18/42	55/134	0.83
HMV	6/42	15/134	0.59

**Table 2:** Causes of acute respiratory failure.

	42 NIV-failure	134 NIV-success	*P* value
Pneumonia	20	29	**0.002**
Congestive heart failure	39	116	0.414
Cardiogenic pulmonary edema	16	48	0.855
Pulmonary embolism	2	3	0.594
Pneumothorax	3	7	0.704

**Table 3:** Numbers and types of acute and chronic comorbidities in the two groups of patients. Numbers in bold font represent the acute comorbidities.

	Total patients population (176 pts) *n* (%)	42 NIV-failure	134 NIV-success	*P*-value* (failure versus success)
Charlson comorbidities				
History myocardial infarction	26 (14.8)	2 (0.8)	24 (17.9)	/
Congestive heart failure	155 (88.1)	39 (92.9)	116 (86.6)	0.414
Peripheral vascular disease	20 (11.4)	5 (11.9)	15 (11.2)	1.00
Cerebrovascular disease	17 (9.7)	5 (11.9)	12 (8.9)	0.558
Chronic pulmonary disease	176 (100)	42 (100)	134 (100)	/
Dementia	25 (14.2)	11 (26.2)	14 (9.9)	**0.02**
Connective tissue disease	3 (1.7)	1 (2.4)	2 (1.5)	/
Peptic ulcer disease	8 (4.5)	1 (2.4)	7 (5.2)	/
Mild liver disease	3 (1.7)	2 (4.8)	1 (0.7)	/
Diabetes without end-organ damage	28 (15.9)	5 (11.9)	23 (17.2)	0.479
Hemiplegia	8 (4.5)	2 (4.8)	6 (4.5)	/
Moderate or severe renal disease	32 (18.2)	12 (28.6)	20 (14.9)	0.065
*Acute on chronic *	*8 (4.5) *	*5 (11.9) *	*3 (2.2) *	
Diabetes with end-organ damage	15 (8.5)	2 (4.8)	13 (9.7)	/
*Glycemia > 200* mg/dL			*5 (3.73) *	
Tumor without metastases	11 (6.2)	1 (2.4)	10 (7.5)	/
Leukemia	1 (0.6)	1 (2.4)	0	/
Lymphoma	3 (1.7)	0	3 (2.2)	/
Moderate or severe liver disease	0	0	0	/
Metastatic solid tumor	6 (3.4)	4 (9.5)	2 (1.5)	/
AIDS	0	0	0	/
Others comorbidities				
Pulmonary embolism	5 (2.8)	2 (4.8)	3 (2.2)	0.594
Obstructive sleep apnea	10 (5.7)	1 (2.4)	9 (6.7)	0.455
Diaphragmatic paralysis	7 (4)	5 (11.9)	2 (1.5)	**0.009**
Fibrothorax	10 (5.7)	1 (2.4)	9 (6.7)	0.455
Bed-ridden syndrome	22 (12.5)	5 (11.9)	17 (12.7)	1.000
Obesity	28 (15.9)	2 (4.8)	26 (19.4)	**0.028**
Pneumothorax	10 (5.7)	3 (7.1)	7 (5.2)	0.704
Kyphoscoliosis	5 (2.8)	0	5 (3.7)	0.340
Pneumonectomy	2 (1.1)	0	2 (1.5)	1.000
*Acute myasthenia *	1 (0.6)	0	1 (0.7)	1.000

**Table 4:** Multivariate analysis. OR = odds ratio. Probability of NIV success increases by 5.6 times for every 1 g/dL increase in albumin serum level, while presence of pneumonia decreases the success probability by 61.8%.

	OR	95% CI		*P*-value
		Inf	Sup	
Gender	0.566	0.243	1.318	0.187
Age	0.969	0.917	1.023	0.255
SAPS score	0.962	0.921	1.006	0.090
Albumin (g/dL)	**5.617**	**2.242**	**14.078**	**0.000**
Charlson index	0.938	0.717	1.227	0.639
Pneumonia	**0.382**	**0.161**	**0.902**	**0.028**
Renal disease	0.760	0.251	2.302	0.628

**Table 5:** Comparison between the different studies concerning COPD and comorbidities, highlighting the main differences in the patients population, methodology, and findings.

	Setting	Age (mean)	Ventilatory treatment	ABG pH PaCO_2_ (mmHg) P/F	APACHE score (II or III)	Pts with more than 1 comorbidity (%)	Criteria to find comorbidity	Predictors of failure treatment and mortality
Dewan et al. (Chest 2000) [21]	Outpatients	66	No ventilatory treatment	—	—	87	Chronic comorbidities	Home oxygen therapy Rate of exacerbations Number of comorbidities
Connors et al. (AMJRCCM 1996) [22]	Hospital wards and ICUs	70	Generically mechanical ventilation (35% of pts)	7.36 56 211	(III) 39	97	Acute comorbidities (causes of COPD exacerbation) and number of chronic ones	APACHE score PaO_2_/FiO_2_, ADL, CHF BMI, cor pulmonale Causes of COPD exacerbations
Moretti et al. (Thorax 2000) [12]	RICUs	70	NIMV (100% of pts at admission) IMV (22% of NIMV failed pts)	7.23 85 —	(II) 22	45 (successful group) 70 (failure group)	Cardiac comorbidities and other complications (pulmonary, metabolic, renal, and gastrointestinal)	Haemodynamic variables Number of associated complications on admission ADL, ABG APACHE II, and age
Scala et al. (Intensive Care Medicine 2004) [9]	Respiratory monitoring unit in a respiratory ward	72	NIMV (100% of pts at admission) IMV (6% of NIMV failed pts)	7.28 78 183	(III) 61	40 (acute comorbidities) 20 (chronic comorbidities)	Acute and chronic comorbidities. Cardiovascular and noncardiovascular ones	Acute cardiovascular comorbidities (influence on NIMV failure) Acute comorbidities and chronic noncardiovascular ones (influence on 6-month mortality)
Nevins and Epstein (Chest 2001) [20]	ICUs	67	IMV (100% of pts)	7.27 68 92	(II) 13	42	Chronic comorbidities and those included in APACHE II	Need of mechanical ventilation APACHE II More than 1 comorbidity
Seneff et al. (JAMA 1995) [10]	ICUs	66	IMV (47% of COPD patients)	—	(III) 57	—	Chronic comorbidities included in APACHE III. Respiratory and nonrespiratory	APACHE III Length of stay before ICU admission
